# Matrix Metalloproteinases and Cardiac Remodelling in Smokers with Acute Coronary Syndrome

**DOI:** 10.3390/ijms27156970

**Published:** 2026-08-03

**Authors:** Amilia Aminuddin, Nazirah Samah, Faridah Mohd Nor, Wan Mohammad Hafiz Wan Razali, Beh Boon Cong, Shawal Faizal Mohamad, Adila A. Hamid, Wafi Khadijah Norhisham, Azizah Ugusman

**Affiliations:** 1 Department of Physiology, Faculty of Medicine, Universiti Kebangsaan Malaysia, Jalan Yaacob Latif, Bandar Tun Razak, Cheras, Kuala Lumpur 56000, Malaysia; p124809@siswa.ukm.edu.my (N.S.); adilahamid@hctm.ukm.edu.my (A.A.H.); p165577@siswa.ukm.edu.my (W.K.N.); dr.azizah@hctm.ukm.edu.my (A.U.); 2 Forensic Unit, Department of Pathology, Faculty of Medicine, Universiti Kebangsaan Malaysia, Jalan Yaacob Latif, Bandar Tun Razak, Cheras, Kuala Lumpur 56000, Malaysia; faridah.nor@ukm.edu.my (F.M.N.); wmhafiz@uitm.edu.my (W.M.H.W.R.); 3 Department of Forensic Pathology, Faculty of Medicine, Sungai Buloh Campus, Universiti Teknologi MARA, Sungai Buloh 47000, Selangor, Malaysia; 4 Cardiology Unit, Hospital Canselor Tuanku Muhriz UKM, Faculty of Medicine, Universiti Kebangsaan Malaysia, Jalan Yaacob Latif, Bandar Tun Razak, Cheras, Kuala Lumpur 56000, Malaysia; dr.behbooncong@hctm.ukm.edu.my (B.B.C.); drshawal81@hctm.ukm.edu.my (S.F.M.)

**Keywords:** acute coronary syndrome, chronic coronary syndrome, matrix metalloproteinase-9, TIMP-3, smoking, echocardiography, Gensini score

## Abstract

Matrix metalloproteinases (MMPs) and their tissue inhibitors (TIMPs) regulate extracellular matrix remodeling and contribute to the pathogenesis of coronary artery disease (CAD). This study examined the associations between MMP-2, MMP-9, and TIMP-3 and coronary atherosclerotic burden and cardiac function among smokers and non-smokers across the CAD spectrum. A total of 180 male participants were enrolled and divided into six groups (*n* = 30 per group): acute coronary syndrome (ACS) smokers, ACS non-smokers, chronic coronary syndrome (CCS) smokers, CCS non-smokers, control smokers, and control non-smokers. Serum MMP-2, MMP-9, and TIMP-3 levels were measured using ELISA. Coronary artery plaque severity was assessed using the Gensini score, while cardiac function was evaluated by echocardiography. MMP-9 and TIMP-3 levels were significantly higher in CCS patients than in controls, regardless of smoking status. MMP-9 showed significant positive correlations with the Gensini score in smokers (*r* = 0.556, *p* < 0.001) and non-smokers (*r* = 0.596, *p* < 0.001) and remained independently associated with coronary plaque severity in both groups. TIMP-3 was independently associated with the Gensini score only in non-smokers (B = 0.667, *p* = 0.031). Higher MMP-9 levels were independently associated with reduced left ventricular ejection fraction and adverse diastolic parameters. Overall, MMP-9 was independently associated with coronary atherosclerotic burden and cardiac dysfunction, supporting its potential as a biomarker of CAD severity. ACS, rather than smoking status, appeared to have a greater influence on cardiac dysfunction.

## 1. Introduction

Cardiovascular disease remains the leading cause of mortality globally, with coronary artery disease (CAD) encompassing a dynamic spectrum ranging from chronic coronary syndrome (CCS) to acute coronary syndrome (ACS). In Malaysia, CAD remains the major cause of death, accounting for 17.0 percent of the 109,155 medically certified deaths in 2020 [1]. This imposes a substantial health and socioeconomic burden on Malaysia by escalating healthcare costs, reducing workforce productivity, and increasing pressure on the national healthcare system.

Matrix metalloproteinases (MMPs), particularly MMP-9, have emerged as key mediators of extracellular matrix (ECM) degradation, contributing to fibrous cap thinning and plaque rupture. Their activity is regulated by tissue inhibitors of metalloproteinases (TIMPs), with TIMP-3 serving as a critical ECM-bound inhibitor that reflects local proteolytic balance. Previous studies have shown that MMP-9 is associated with the severity of atherosclerotic plaque [2,3,4]. In CAD, chronic myocardial ischemia leads to increased ventricular wall stress, inflammation, and myocardial injury, which subsequently lead to structural and functional changes in the heart, including ventricular hypertrophy, fibrosis, dilation, and impaired cardiac function [5]. Cigarette smoking, a major modifiable cardiovascular risk factor, has been associated with an enhanced proteolytic environment through activation of inflammatory signalling pathways in both the heart and vessels [6]. This has been associated with enhanced MMP-9 expression and sustained ECM degradation in both structures.

Our previous paper showed that MMP-9 was increased among ACS and CCS smokers; however, whether MMP-9 is simultaneously associated with coronary plaque burden and cardiac remodeling across different stages of CAD and whether these relationships differ according to smoking status remain poorly understood [7]. Addressing this knowledge gap may improve our understanding of the molecular mechanisms linking vascular pathology with myocardial dysfunction. Therefore, this study aimed to investigate the associations between MMP-2, MMP-9, and TIMP-3 and coronary atherosclerotic burden, assessed using the Gensini score, and with echocardiographic measures of systolic and diastolic function in smokers and non-smokers across the CAD spectrum.

## 2. Results

### 2.1. Demographic and Clinical Characteristics of Patients

A total of 180 subjects were recruited for this study, with 30 participants allocated to each study group. Comparisons of demographic and clinical characteristics revealed several significant intergroup differences (Table 1). Age differed significantly across the groups (*p* = 0.001), with CCS non-smokers being significantly older than control non-smokers (*p* < 0.05). Hypertension prevalence also varied significantly (*p* < 0.001), as CCS patients exhibited higher rates than controls among both smokers (*p* < 0.05) and non-smokers (*p* < 0.001). Significant variation was also observed in dyslipidaemia prevalence (*p* = 0.002), whereby CCS patients demonstrated higher rates compared with ACS patients in both smoking and non-smoking groups (*p* < 0.05 for both). Conversely, a family history of CAD was more frequently reported among controls than among CCS patients in both smokers and non-smokers (*p* < 0.05 for both). No significant differences were identified for diabetes mellitus. For cholesterol-lowering therapy, among smokers, control participants were more likely to receive cholesterol-lowering medications than ACS patients (*p* < 0.05). Likewise, within the non-smoking group, the use of cholesterol-lowering agents was significantly higher among controls compared with both ACS and CCS patients (*p* < 0.05 for both). No significant differences were observed for other types of drugs across the groups, such as ACE inhibitors, angiotensin II inhibitors, anticoagulant agents, antiplatelet agents, beta blockers and calcium-channel blockers. Detailed baseline medication characteristics have been reported previously in our earlier publication using the same study cohort [7]. Cholesterol-lowering medication, age, hypertension, dyslipidaemia, and family history of CAD were incorporated as covariates in subsequent analyses [8,9].

For Gensini scores, a significant difference was observed among the groups after covariate adjustment (*p* < 0.001). In both smokers and non-smokers, the Gensini scores for the CCS and ACS groups were significantly higher than those of the control group (*p* < 0.001), indicating greater severity of atherosclerosis in both disease groups. Among non-smokers, the Gensini score was significantly higher in the ACS group compared with the CCS group (*p* = 0.028), suggesting a greater atherosclerotic burden in ACS.

### 2.2. Protein Level Analysis

Table 2 summarizes the MMP-2, MMP-9 and TIMP-3 levels across the groups. After adjustment for covariates, MMP-2 protein levels did not differ significantly between the study groups. For MMP-9, the protein levels differed significantly across the study groups prior to covariate adjustment. This significance persisted following analysis of covariance (ANCOVA) adjustment for covariates (*p* < 0.001), suggesting sustained intergroup differences. In the CCS subgroup, smokers demonstrated significantly elevated MMP-9 levels compared with non-smokers (*p* = 0.036). Furthermore, in both smoking and non-smoking participants, MMP-9 concentrations were significantly higher in the ACS and CCS groups than in the control group (*p* < 0.001 for both).

TIMP-3 protein levels showed significant differences across the study groups prior to covariate adjustment, and the differences remained significant after adjustment for covariates (*p* < 0.001). Among smokers, patients with CCS exhibited significantly higher TIMP-3 levels compared with controls (*p* < 0.001). Similarly, in the non-smoking group, TIMP-3 concentrations were significantly elevated in both ACS and CCS patients relative to controls (*p* < 0.001 for both).

### 2.3. Relationship Between Protein Levels and Gensini Score Among Smokers and Non-Smokers

Table 3 shows the relationship analysis of protein biomarkers with the Gensini score among smokers and non-smokers. Among smokers, Spearman correlation analysis showed that MMP-2 was not significantly associated with the Gensini score (*r* = 0.052, *p* = 0.624), whereas MMP-9 (*r* = 0.556, *p* < 0.001) and TIMP-3 (*r* = 0.310, *p* = 0.003) demonstrated significant moderate positive correlations. However, multiple linear regression analysis identified only MMP-9 (B = 0.031, *p* = 0.024) as a significant predictor of the Gensini score, suggesting its dominant contribution to atherosclerotic plaque progression among smokers.

Among non-smokers, Spearman correlation analysis demonstrated that MMP-2 (*r* = 0.228, *p* = 0.030), MMP-9 (*r* = 0.596, *p* < 0.001), and TIMP-3 (*r* = 0.369, *p* < 0.001) were significantly and positively correlated with the Gensini score, although with weak-to-moderate strength. Multiple linear regression analysis further identified MMP-2 (B = 0.055, *p* = 0.032), MMP-9 (B = 0.032, *p* = 0.001), and TIMP-3 (B = 0.667, *p* = 0.031) as significant predictors of the Gensini score.

### 2.4. Relationship Between Protein Levels of MMP-2, MMP-9 and TIMP-3 with Echocardiography Parameters (LVEF, E, E/A, Av E/e′ and EDT)

Table 4 shows the mean values of systolic function (LVEF) and diastolic function (E velocity, E/A ratio, Av E/e′, and EDT) among the ACS, CCS and control groups according to smoking status after covariate adjustment. A significant difference in LVEF was observed among the groups (*p* = 0.005). Results showed that ACS smokers had a significantly lower LVEF compared with control smokers (62.97 ± 1.23 vs. 57.20 ± 1.11, *p* = 0.007), suggesting that greater disease severity may reduce left ventricular systolic function.

For E velocity, the overall effect remained significantly different among groups (*p* < 0.001). In both smokers and non-smokers, ACS had significantly higher E velocity compared with control (ACS smokers: 0.805 ± 0.016 vs. control smokers: 0.661 ± 0.017, *p* = 0.004; ACS non-smokers: 0.798 ± 0.016 vs. control non-smokers: 0.673 ± 0.017, *p* = 0.039), suggesting that greater disease severity is associated with increased E velocity. For the E/A ratio, the overall effect also remained significantly different among groups (*p* = 0.023). Paired comparison analysis showed that ACS non-smokers had a higher E/A ratio compared with control smokers, which differed slightly from the results before covariate adjustment.

For Av E/e′, the overall effect remained significantly different among groups (*p* < 0.001). Among non-smokers, the ACS group showed a significantly higher Av E/e′ compared with controls (10.32 ± 0.32 vs. 7.89 ± 0.33, *p* < 0.001), suggesting that greater disease severity may increase Av E/e′. For EDT, the overall effect was not significantly different among groups (*p* = 0.270).

Table 5 shows the relationship analysis of protein biomarkers with echocardiogram parameters among smokers and non-smokers. For LVEF, among smokers, Spearman correlation analysis showed that MMP-2 (*r* = 0.030, *p* = 0.777) and TIMP-3 (*r* = −0.118, *p* = 0.267) were not significantly associated with LVEF, whereas MMP-9 (*r* = −0.267, *p* < 0.001) demonstrated a significant weak negative correlation. Multiple linear regression analysis further identified only MMP-9 (B = −0.005, *p* = 0.038) as a significant predictor of LVEF. In non-smokers, Spearman correlation analysis showed that none of the protein biomarkers were significantly associated with LVEF. However, multiple linear regression revealed that MMP-9 (B = −0.005, *p* = 0.006) remained a significant predictor of LVEF.

For E velocity, among smokers, Spearman correlation analysis showed no significant associations with any of the biomarkers. However, multiple linear regression identified MMP-2 (B = 0.001, *p* = 0.044) as a significant positive predictor of E velocity. Among non-smokers, both MMP-2 (*r* = 0.209, *p* = 0.048) and MMP-9 (*r* = 0.259, *p* = 0.014) showed significant weak positive correlations with E velocity, whereas TIMP-3 (*r* = 0.040, *p* = 0.711) was not significant. Nevertheless, only MMP-9 (B = 0.000, *p* = 0.022) remained a significant positive predictor in the regression model.

For the E/A ratio, both Spearman correlation and multiple linear regression analyses showed no significant associations with any biomarkers in both smokers and non-smokers. For Av E/e′, no significant associations were observed among smokers. In contrast, among non-smokers, MMP-2 (*r* = 0.247, *p* = 0.019), MMP-9 (*r* = 0.468, *p* < 0.001), and TIMP-3 (*r* = 0.261, *p* = 0.013) showed significant weak-to-moderate positive correlations with Av E/e′. However, multiple linear regression identified only MMP-9 (B = 0.002, *p* = 0.010) as a significant positive predictor. For EDT, no significant associations were observed among smokers. However, in non-smokers, multiple linear regression identified MMP-9 (B = −0.025, *p* = 0.025) as a significant negative predictor of EDT.

## 3. Discussion

Our findings showed that MMP-9 and TIMP-3 levels remained significantly elevated in CCS patients compared with controls after covariate adjustment, in both smoking and non-smoking groups, whereas no significant differences were observed for MMP-2. As the intergroup differences in MMPs and TIMP-3 levels have been previously discussed [7], the current discussion focuses on their associations with angiographic severity and echocardiographic parameters. To our knowledge, few studies have comprehensively evaluated the relationships between MMP-2, MMP-9, TIMP-3, coronary plaque burden, and both systolic and diastolic echocardiographic parameters within the same cohort of smokers and non-smokers with CAD.

### 3.1. The Association Between MMP-2, MMP-9 and TIMP-3 with Gensini Scores

Our findings showed that, among smokers, MMP-9 had a significant positive association with Gensini scores, whereas among non-smokers, MMP-2 and MMP-9 were significantly associated with Gensini scores. These findings are consistent with previous studies, but our research further extends these observations to the smoker population [10,11]. Experimental studies have shown that nicotine exposure stimulates the α1nAChR receptor, which is associated with increased inflammatory pathways such as ERK 1/2 and increased MMP-9 expression and atherosclerotic plaque burden [7,12]. Among non-smokers with CAD, Tanindi et al. [10] reported that elevated MMP-9 levels were associated with more severe stenosis of the left anterior descending artery and higher angiographic Gensini scores in patients with CAD. Similarly, Liu et al. [11] demonstrated a strong positive correlation between MMP-9 levels and the Gensini score.

In this study, TIMP-3 was also positively associated with Gensini score. A similar finding was observed in the study by Han et al. [13], whereby the researchers found that TIMP-22 was significantly correlated with angiographically assessed severity and the number of vessels involved in coronary artery disease. Expression levels of human TIMP-3 were also increased in normal coronary arterial segments of cases with coronary heart disease (CHD), suggesting a protective role of TIMP-3 in the development of atherosclerosis [14]. Besides this, TIMP-4 has been shown to exert protective effects against plaque deposition in the abdominal aorta, independent of plasma cholesterol levels [15]. In contrast, a previous study found that TIMP was negatively correlated with cardiovascular disease (CVD) risk factors such as systolic blood pressure (BP) and smoking among young subjects [16]. This divergence in findings underscores the complexity of the relationship between TIMPs, atherosclerosis, and CAD [17]. Nevertheless, the positive associations between TIMP-3 and the severity of atherosclerotic plaque in our study may indicate that, as a natural inhibitor of MMP-2 and MMP-9, TIMP-3 may reflect compensatory regulation in response to increased MMP activity during atherosclerotic progression. Elevated TIMP-3 levels could indicate active vascular remodelling and ongoing inflammatory processes associated with CAD severity [17].

Overall, our findings extend previous reports by demonstrating that the relationship between MMPs, TIMPs and angiographic severity remains evident after stratification according to smoking status. The integration of biomarker assessment with angiographic evaluation may therefore improve risk stratification and provide additional prognostic value in patients with CAD.

### 3.2. Markers of Systolic Function (LVEF) Between the Groups and Their Association with MMP-2, MMP-9 and TIMP-3 Levels

Our findings showed that LVEF was significantly reduced in ACS smokers compared to the control and CCS groups, but no significant difference was observed between ACS smokers and ACS non-smokers. This finding suggests that reduced left ventricular systolic function is more strongly associated with ACS than with smoking status. This finding is also consistent with previous studies reporting that patients with ACS commonly experience reduced left ventricular systolic function due to myocardial cell death, inflammatory responses, and degradation of the ECM [18,19]. Myocardial necrosis and apoptosis trigger the release of pro-inflammatory cytokines such as TNF-α, IL-1β, and IL-6 [20,21]. These inflammatory processes activate fibroblasts, macrophages, and neutrophils, which increase the production of MMPs [22]. Elevated MMP activity has been associated with ECM degradation, leading to ventricular wall thinning and reduced structural stability and elasticity [23]. Progressive ECM degradation may contribute to increased ventricular stiffness and reduced compliance, reducing ventricular dilation [24]. These structural changes may contribute to reduced preload, myocardial contractility, stroke volume, and LVEF [25]. In addition, smoking may further worsen systolic function by increasing inflammatory mediators and MMP activity, particularly MMP-9 [12,26]. This is supported by our finding, which showed a significant negative association between MMP-9 and LVEF, indicating a significant role for MMP-9 as a biological marker associated with reduced systolic function. This observation was also consistent with previous clinical studies reporting an inverse association between circulating MMP-9 levels and LVEF in patients with ischemic heart disease. Hamed and Fattah [27] reported that early post-infarction MMP-9 levels were negatively correlated with LVEF. Similarly, Kelly et al. [28] demonstrated that higher peak MMP-9 levels were associated with more severe left ventricular dysfunction during hospitalization in patients with CAD. Overall, the lower LVEF observed in ACS reflects the impact of disease severity on systolic function, which may be mediated by increased MMP-9 activity.

### 3.3. Markers of Diastolic Function (E, E/A Ratio, Av E/e′, EDT) Between Groups

Generally, the present findings suggest that smoking status did not significantly influence the severity of diastolic dysfunction among ACS patients, as no significant differences were observed between ACS smokers and ACS non-smokers. Instead, the presence of ACS itself appeared to be more strongly associated with diastolic functional alterations, as evidenced by the differences in E velocity, Av E/e′ and E/A ratio. This observation may indicate that ischemic myocardial injury associated with ACS plays a more dominant role in determining diastolic impairment than smoking status alone. Ischemia leads to cardiomyocyte death, which is followed by inflammation and scar formation and subsequently leads to increased ventricular stiffness and reduced compliance [29,30]. There is impaired myocardial relaxation, and left ventricular filling pressure rises [31,32].

Our study showed that E velocity was higher among ACS patients when compared to the control and CCS groups. Early diastolic E velocity is influenced by LV relaxation and LA pressure [33,34]. In patients with impaired LV relaxation, elevated LA pressure may compensate by increasing E velocity, resulting in a pseudonormal or restrictive filling pattern [34]. Therefore, the higher E velocity observed in ACS likely reflects elevated LA pressure [35,36]. It is worth noting that the value remains within the normal physiological range, suggesting subtle alterations in left ventricular filling dynamics and possible early diastolic impairment associated with acute ischemia. The higher E velocity was accompanied by a higher E/A ratio in ACS patients. A higher value may reflect the early stage of moderate diastolic dysfunction (pseudonormalization) [37,38].

Our study also showed that ACS causes higher Av E/e′. Average E/e′ is a well-established non-invasive marker of LV filling pressure and diastolic dysfunction [39,40]. The higher average E/e′ observed in ACS patients therefore suggests elevated LV filling pressure and impaired ventricular relaxation. This finding is consistent with previous studies demonstrating that increased E/e′ predicts adverse cardiovascular outcomes after acute myocardial infarction [41].

For EDT, our study showed no differences between the groups. A similar finding was observed in a previous study by Kenawy et al. [42], which reported no significant difference in EDT values between ST-elevation myocardial infarction (STEMI) patients with and without left ventricular dysfunction. The values of all groups remained within the normal reference range (150–240 ms) [38,43]. Prolonged EDT (>240 ms) is associated with slower ventricular filling and reduced left ventricular compliance, indicating impaired diastolic relaxation. In contrast, shortened EDT (<150 ms) reflects increased left ventricular filling pressure and is characteristic of a restrictive filling pattern [38,44]. Altered EDT may occur in advanced disease and explains why the value is still normal in our study.

### 3.4. The Association Between MMP-2, MMP-9 and TIMP-3 with E, Av E/e′, E/A Ratio and EDT

Although ACS was associated with alterations in diastolic function, the extent to which these changes are related to extracellular matrix remodelling remains unclear. Therefore, we further examined the associations between MMP-2, MMP-9, TIMP-3 and diastolic function parameters. Our study showed that MMP-2, MMP-9 and TIMP-3 had significant associations with diastolic function parameters such as E velocity and Av E/e′, which were more pronounced among non-smokers. Linssen et al. [45] further reported that elevated serum MMP-2 levels are associated with increased left atrial volume index, reflecting higher filling pressure. Increased MMP-9 has been associated with shorter EDT in patients with ischemic heart disease [46]. These findings suggest a role for MMP-2 and MMP-9 in modulating diastolic function. Increased MMP-2 activity has been associated with extracellular matrix degradation, interstitial fibrosis, and increased myocardial stiffness [47]. Similarly, experimental studies suggest that increased MMP-9 activity promotes matrix degradation and vascular permeability via Vascular Endothelial Growth Factor (VEGF), leading to provisional matrix formation and increased ventricular stiffness. This results in elevated left ventricular filling pressure and left atrial pressure, thereby increasing the LA–LV pressure gradient and E velocity [18,48,49]. Animal studies further support this, showing that *MMP-9* gene deletion improves diastolic relaxation [50].

No significant associations were observed for E/A ratio. Other biomarkers such as Brain Natriuretic Peptide are more well-established markers of left ventricular filling pressure and are closely associated with mitral inflow patterns, including the E/A ratio [51]. In addition, biomarkers of inflammation and cardiac wall stress, such as galectin-3 and soluble ST2 (sST2), have also been linked to indices of diastolic dysfunction [52,53]. This suggests that the E/A ratio may be influenced by other biomarkers more directly related to diastolic function.

There were several limitations in this study. First, this study included only male participants to minimise biological variability associated with sex hormones and sex-specific cardiovascular physiology. Consequently, the findings may not be directly generalisable to female patients. Future studies should include both sexes to determine whether the observed associations between MMPs, smoking, coronary plaque burden, and cardiac remodelling differ according to sex. Secondly, although only cholesterol-lowering therapy differed significantly between the study groups and was included as a covariate, we acknowledge that cardiovascular medications may influence inflammatory biomarkers and cardiac function. Therefore, residual confounding related to medication use cannot be completely excluded. Thirdly, although blood samples were collected within the first 24 h of admission and before coronary angiography/PCI, the exact interval between symptom onset and blood collection was not available. Because MMP-9 concentrations vary over time following myocardial infarction, this may have contributed to inter-individual variability. Future prospective studies should standardize blood sampling according to symptom onset.

## 4. Methods

Ethical approval for this study was granted by the Centre for Research and Instrumentation Management (CRIM), Universiti Kebangsaan Malaysia (UKM PPI/111/8/JEP-2022-112; 29 March 2022), and all procedures were conducted in accordance with the Declaration of Helsinki. Patient recruitment and sample collection were conducted at Hospital Canselor Tuanku Muhriz (HCTM), Universiti Kebangsaan Malaysia. All patients were required to give informed consent. Samples were taken from male patients aged ≥18 years who were undergoing computed tomography (CT) or coronary angiography for suspected or diagnosed coronary artery disease (CAD). Only male participants were included to minimise biological variability related to sex differences in MMP expression, hormonal influences, and cardiovascular remodelling. This approach was adopted to reduce potential confounding when evaluating the associations between MMPs, coronary plaque severity, and cardiac function. Patients were divided into six groups (*n* = 30 per group): ACS smokers, ACS non-smokers, CCS smokers, CCS non-smokers, control smokers, and control non-smokers. The sample size was calculated using the PS Sample Size Calculator. Based on the study by Sivaraman et al. (2014) [54], the mean difference in MMP-9 levels between smokers and non-smokers among patients with acute myocardial infarction was 15 ng/mL, with a standard deviation of 20 ng/mL. Using a significance level (α) of 0.05 and a study power of 80%, the calculated sample size was around 30 participants per group.

ACS was defined as chest pain with ECG changes and elevated troponin I. CCS was defined as chest pain without ACS and with >50% stenosis seen on angiogram/CT. Control subjects were those with non-significant plaque obstruction (<50% stenosis seen on angiogram/CT). Smokers were defined as individuals who had smoked more than 10 pack-years [55]. For all groups, those with a history of chronic diseases (lung disease/kidney disease/cancer/chronic inflammatory diseases and others) were excluded. Clinical data, smoking history, and medication records were collected from patients or retrieved from hospital records, while smoking exposure was quantified using pack-year calculations.

For patients with ACS, venous blood samples were collected within the first 24 h of hospital admission and prior to coronary angiography/PCI. Serum was processed and stored at −80 °C until analysis. Protein levels of MMP-2, MMP-9, and TIMP- 3 were measured using commercially available human ELISA kits (Elabscience Biotechnology Inc., Houston, TX, USA) according to the manufacturer’s protocols [56]. The MMP-2 ELISA kit (Catalog No.: E-EL-H1445) exhibited intra-assay and inter-assay coefficients of variation (CVs) of 4.13–5.66% and 3.37–8.33%, respectively, with a detection range of 0.78–50 ng/mL. The MMP-9 ELISA kit (Catalog No.: E-EL-H6075) showed intra-assay and inter-assay CVs of 4.04–5.76% and 6.48–7.10%, respectively, with a detection range of 0.16–10 ng/mL. The TIMP 3 ELISA kit (Catalog No.: E-EL-H1454) demonstrated intra-assay and inter-assay CVs of 4.31–6.27% and 6.78–7.37%, respectively, with a detection range of 62.5–4000 pg/mL. Serum samples were centrifuged, diluted prior to analysis, and assayed in duplicate alongside serially diluted standards to generate standard curves. Absorbance was measured at 450 nm using a microplate reader (Molecular Devices, LLC, San Jose, CA, USA), and protein concentrations were determined based on the respective standard curves.

### 4.1. Gensini Score

Computed tomography (CT) or coronary angiography results from each patient were used to determine the Gensini score. The Gensini scoring system was applied to assess the severity of coronary artery stenosis, as described by Nurkalem et al. [57]. This system assigns scores based on the degree of luminal narrowing and the anatomical location of the stenosis in the coronary arteries. The percentage of stenosis severity was graded with scores according to the level of luminal narrowing. Each score was then multiplied by a weighting factor corresponding to the location of the affected coronary artery segment. The total Gensini score was calculated by summing all the calculated scores.

### 4.2. Echocardiography Parameters

Transthoracic echocardiography results from each patient were used to evaluate left ventricular (LV) systolic and diastolic function. The parameters analysed included left ventricular ejection fraction (LVEF), early mitral inflow velocity (E velocity), ratio of E velocity to atrial velocity (E/A), average ratio of E velocity to early diastolic mitral annular velocity (e′) (Av E/e′) and E-wave deceleration time (EDT) [58].

### 4.3. Statistical Analysis

All statistical analyses were conducted using IBM SPSS Statistics software version 30. The normality of the data distribution was evaluated with the Shapiro–Wilk test. Variables that did not meet the assumption of normality were subjected to log_10_ transformation before further analysis. Group differences in protein concentrations were first examined using one-way analysis of variance (ANOVA) without adjustment for covariates. To control for possible confounding effects, analysis of covariance (ANCOVA) was then conducted with age, hypertension, dyslipidaemia, family history of coronary artery disease (CAD), and use of lipid-lowering medication included as covariates. These covariates were incorporated into the model as fixed-effect predictors. Hypertension, dyslipidaemia, family history of CAD and medication use were entered as categorical variables, while age was included as a continuous variable. A *p*-value of less than 0.05 was considered statistically significant. Data are expressed as mean ± standard error of the mean (SEM) on their original scale. Subsequently, Spearman correlation, multiple linear regression and hierarchical regression analyses were performed to evaluate the pattern and strength of the associations between echocardiographic parameters and the protein levels of MMP-2, MMP-9, and TIMP-3 among smokers and non-smokers, with adjustment for relevant covariates.

## 5. Conclusions

Overall, these findings highlight the potential role of MMPs and TIMPs, particularly MMP-9, as biomarkers associated with coronary severity and cardiac dysfunction in CAD. The integration of biomarker assessment with echocardiographic and angiographic evaluations may therefore improve cardiovascular risk stratification and provide additional prognostic value in patients with coronary artery disease. Although smoking has been associated with activation of inflammatory and atherogenic pathways, the present findings suggest that ACS was more strongly associated with systolic and diastolic dysfunction than smoking status alone.

## Figures and Tables

**Table 1:** Demographic and clinical characteristics of patients.

Characteristics	Control		CCS		ACS		*p*-Value
	Non-Smokers (*n* = 30)	Smokers (*n* = 30)	Non-Smokers (*n* = 30)	Smokers (*n* = 30)	Non-Smokers (*n* = 30)	Smokers (*n* = 30)	
Age (years)	50.77 ± 2.12	49.03 ± 2.07	60.87 ± 1.72 ^a^	53.50 ± 1.98	56.17 ± 2.47	51.83 ± 1.98	0.001 *
BMI (kg/m^2^)	26.54 ± 0.85	26.09 ± 0.63	26.41 ± 0.70	25.20 ± 0.54	26.76 ± 0.82	25.64 ± 0.67	0.703
Heart rate (bpm)	64.5 ± 1.70	70.20 ± 2.05	78.97 ± 2.03 ^b^	75.0 ± 2.71	78.07 ± 2.82 ^c^	65.77 ± 1.57	<0.001 *
Hypertension, *n* (%)	11 (36.7%)	12 (40%)	27 ^d^ (90%)	20 ^e^ (66.7%)	23 (76.7%)	16 (53.3%)	<0.001 *
Diabetes mellitus, *n* (%)	9 (30%)	9 (30%)	16 (53.3%)	13 (43.3%)	14 (46.7%)	8 (26.7%)	0.183
Dyslipidaemia, *n* (%)	19 (63.3%)	15 (50%)	24 ^f^ (80%)	20 ^g^ (66.7%)	12 (40%)	10 (33.3%)	0.002 *
Family history of CAD, *n* (%)	10 (33.3%)	11 ^h^ (36.7%)	3 (10%)	3 (10%)	5 (16.7%)	5 (16.7%)	0.029 *
Cholesterol-lowering drugs, *n* (%)	23 (76.7%)	18 (60.0%)	14 ^i^ (46.7%)	13 ^j^ (43.3%)	12 ^k^ (40.0%)	10 ^l,m^ (33.3%)	0.011 *
Pack-years	-	20.44 ± 9.41	-	21.61 ± 10.25	-	26.06 ± 12.64	0.19
Gensini score (after covariate adjustment)	2.09 ± 1.17	2.20 ± 1.17	23.12 ± 1.17 ^n,o^	37.67 ± 1.16 ^p,q^	46.34 ± 1.17 ^r,s,t^	40.64 ± 1.17 ^u,v^	<0.001 ^δ^

* *p* < 0.05, significant. Data for age, BMI, heart rate, and Gensini scores are presented as mean ± SEM. Data for hypertension, diabetes mellitus, dyslipidaemia, and family history of CAD are presented as *n*, the number of patients. Data for pack-years are presented as mean ± SD. Post hoc analysis: ^a^ *p* < 0.05, CCS NS vs. control NS; ^b^ *p* < 0.001, CCS non-smokers vs. control NS; ^c^ *p* < 0.001, ACS NS vs. control NS; ^d^ *p* < 0.001, CCS NS vs. control NS; ^e^ *p* < 0.05, CCS smokers vs. control smokers; ^f^ *p* < 0.05, CCS NS vs. ACS NS; ^g^ *p* < 0.05, CCS smokers vs. ACS smokers; ^h^ *p* < 0.05, control smokers vs. CCS smokers; ^i^ *p* < 0.05, CCS NS vs. control NS; ^j^ *p* < 0.05, CCS smokers vs. control NS; ^k^ *p* < 0.05, ACS NS vs. control NS; ^l^ *p* < 0.05, ACS smokers vs. control smokers; ^m^ *p* < 0.001, ACS smokers vs. control NS; ^n^ *p* < 0.001, CCS NS vs. control NS; ^o^ *p* < 0.001, CCS NS vs. control smokers; ^p^ *p* < 0.001, CCS smokers vs. control NS; ^q^ *p* < 0.001, CCS smokers vs. control smokers; ^r^ *p* < 0.001, ACS NS vs. control NS; ^s^ *p* < 0.001, ACS NS vs. control smokers; ^t^ *p* = 0.028, ACS NS vs. CCS NS; ^u^ *p* < 0.001, ACS smokers vs. control NS; ^v^ *p* < 0.001, ACS smokers vs. control smokers; ^δ^ *p* = *p*-value after adjustment for covariates including age, hypertension, dyslipidaemia, family history of CAD, and cholesterol-lowering medication; NS = non-smokers.

**Table 2:** Levels of MMPs and TIMP-3 among the subjects.

Protein	Control + NS	Control + S	CCS + NS	CCS + S	ACS + NS	ACS + S	*p*-Value
MMP-2 (ng/mL)	222.84 ± 1.1	242.10 ± 1.1	276.69 ± 1.1	287.08 ± 1.1	285.76 ± 1.1	233.35 ± 1.1	0.084 ^δ^
MMP-9 (ng/mL)	79.43 ± 1.2	87.70 ± 1.2	197.24 ± 1.2 ^a,b^	364.75 ± 1.2 ^c,d,e^	411.15 ± 1.2 ^f,g,h^	376.70 ± 1.2 ^i,j,k^	<0.001 ^δ^
TIMP-3 (ng/mL)	14.55 ± 1.1	16.94 ± 1.1	28.05 ± 1.1 ^l,m^	26.98 ± 1.1 ^n,o^	22.23 ± 1.1 ^p^	21.33 ± 1.1 ^q^	<0.001 ^δ^

Data are mean ± SEM. ^a^ *p* < 0.001, CCS NS vs. control NS; ^b^ *p* = 0.004, CCS NS vs. control smokers; ^c^ *p* < 0.001, CCS smokers vs. control NS; ^d^ *p* < 0.001, CCS smokers vs. control smokers; ^e^ *p* = 0.036, CCS smokers vs. CCS NS; ^f^ *p* < 0.001, ACS NS vs. control NS; ^g^ *p* < 0.001, ACS NS vs. control smokers; ^h^ *p* = 0.005, ACS NS vs. CCS NS; ^i^ *p* < 0.001, ACS smokers vs. control NS; ^j^ *p* < 0.001, ACS smokers vs. control smokers; ^k^ *p* = 0.035, ACS smokers vs. CCS NS; ^l^ *p* < 0.001, CCS NS vs. control NS; ^m^ *p* < 0.001, CCS NS vs. control smokers; ^n^ *p* < 0.001, CCS smokers vs. control NS; ^o^ *p* < 0.001, CCS smokers vs. control smokers; ^p^ *p* = 0.004, ACS NS vs. control NS; ^q^ *p* = 0.010, ACS smokers vs. control NS; NS = non-smokers; ^δ^ *p* = *p*-value after adjustment for covariates including age, hypertension, dyslipidaemia, family history of CAD, and cholesterol-lowering medication.

**Table 3:** Spearman correlation and multiple linear regression analysis of biomarkers with the Gensini score among smokers and non-smokers.

		MMP-2				MMP-9				TIMP-3			
		*r* (*p*)	B, β (*p*)	95% CI	R^2^, Adjusted R^2^	*r* (*p*)	B, β (*p*)	95% CI	R^2^, Adjusted R^2^	*r* (*p*)	B, β (*p*)	95% CI	R^2^, Adjusted R^2^
Gensini Score	Smokers	0.052 (0.624)	−0.017, −0.046 (0.680)	−0.101 to 0.067	0.011, −0.61	0.556 (<0.001) *	0.031, 0.252 (0.024) *	0.005 to 0.057	0.069, 0.01	0.310 (0.003) *	0.774, 0.183 (0.097)	−0.143 to 1.691	0.041, −0.28
	Non-Smokers	0.228 (0.030) *	0.055, 0.227 (0.032) *	0.005 to 0.105	0.227, 0.171	0.596 (<0.001) *	0.032, 0.326 (<0.001) *	0.012 to 0.052	0.281, 0.229	0.369 (<0.001) *	0.667, 0.220 (0.031) *	0.064 to 1.270	0.227, 0.171

* *p* < 0.05, significant. *r*, Spearman correlation coefficient; B, unstandardized coefficient of multiple linear regression.

**Table 4:** LVEF, E velocity, E/A ratio, Av E/e′ and EDT in the ACS, CCS and control groups.

Groups		LVEF	E Velocity	E/A Ratio	Av E/e′	EDT
Controls	Non-Smokers	61.58 ± 1.23	0.673 ± 0.017	1.058 ± 0.051	7.89 ± 0.33	211.15 ± 7.37
	Smokers	62.97 ± 1.23	0.661 ± 0.017	0.958 ± 0.050	8.29 ± 0.32	203.76 ± 7.26
CCS	Non-Smokers	62.23 ± 1.26	0.697 ± 0.017	1.059 ± 0.052	8.99 ± 0.34	196.63 ± 7.51
	Smokers	59.22 ± 1.13	0.728 ± 0.016	1.141 ± 0.049	8.80 ± 0.32	198.16 ± 7.08
ACS	Non-Smokers	60.11 ± 1.16	0.798 ± 0.016 ^d,e^	1.175 ± 0.049 ^h^	10.32 ± 0.32 ^i,j,k^	186.04 ± 7.16
	Smokers	57.20 ± 1.11 ^a,b,c^	0.805 ± 0.016 ^f,g^	1.160 ± 0.049	9.56 ± 0.32 ^l^	204.43 ± 7.15
*p*-value		0.005	<0.001	0.023	<0.001	0.270

Data are presented as mean ± SEM. ^a^ *p* = 0.009, ACS smokers vs. control NS; ^b^ *p* = 0.007, ACS smokers vs. control smokers; ^c^ *p* = 0.050, ACS smokers vs. CCS NS; ^d^ *p* = 0.039, ACS NS vs. control NS; ^e^ *p* = 0.010, ACS NS vs. control smokers; ^f^ *p* = 0.019, ACS smokers vs. control NS; ^g^ *p* = 0.004, ACS smokers vs. control smokers; ^h^ *p* = 0.041, ACS NS vs. control smokers; ^i^ *p* < 0.001, ACS NS vs. control NS; ^j^ *p* < 0.001, ACS NS vs. control smokers; ^k^ *p* = 0.014, ACS NS vs. CCS smokers; ^l^ *p* = 0.007, ACS smokers vs. control NS. *p* = *p*-value after adjustment for covariates including age, hypertension, dyslipidemia, family history of CAD, and cholesterol-lowering medication.

**Table 5:** Spearman correlation and multiple linear regression analysis of biomarkers with E velocity, E/A ratio, Av E/e′ and EDT among smokers and non-smokers.

		MMP-2				MMP-9				TIMP-3			
		*r* (*p*)	B, β (*p*)	95% CI	R^2^, Adjusted R^2^	*r* (*p*)	B, β (*p*)	95% CI	R^2^, Adjusted R^2^	*r* (*p*)	B, β (*p*)	95% CI	R^2^, Adjusted R^2^
Smokers	LVEF	0.030 (0.777)	0.001, 0.017 (0.881)	−0.013 to 0.015	0.025, −0.046	−0.267 (<0.001) *	−0.005, −0.230 (0.038) *	−0.009 to −0.001	0.074, 0.007	−0.118 (0.267)	−0.082, −0.121 (0.270)	−0.229 to 0.065	0.039, −0.031
	E velocity	−0.180 (0.090)	0.000, −0.219 (0.044) *	−0.000199 to 0.000199	0.325, 0.106	0.096 (0.367)	3.133 × 10^−5^, 0.060 (0.584)	−0.000168 to 0.000230	0.253, 0.064	−0.057 (0.597)	−0.002, −0.095 (0.379)	−0.006 to 0.002	0.069, 0.002
	E/A ratio	−0.067 (0.529)	0.000, −0.073 (0.480)	−0.000199 to 0.000199	0.178, 0.119	0.130 (0.222)	6.455 × 10^−5^, 0.080 (0.436)	−0.000134 to 0.000263	0.179, 0.120	0.012 (0.913)	0.001, 0.027 (0.789)	−0.005 to 0.007	0.174, 0.114
	Av E/e′	−0.088 (0.410)	7.088 × 10^−5^, 0.004 (0.970)	−0.0039 to 0.0040	0.111, 0.047	−0.032 (0.767)	−1.042 × 10^−5^, −0.002 (0.986)	−0.0020 to 0.0020	0.111, 0.047	−0.076 (0.475)	−0.010, −0.049 (0.643)	−0.052 to 0.032	0.113, 0.049
	EDT	0.000 (1.000)	−0.001, −0.003 (−0.974)	−0.083 to 0.081	0.119, 0.056	−0.032 (0.764)	0.005, 0.040 (0.705)	−0.021 to 0.031	0.121, 0.057	−0.078 (0.464)	−0.411, −0.095 (0.364)	−1.306 to 0.484	0.128, 0.065
Non-Smokers	LVEF	−0.039 (0.717)	0.003, 0.074 (0.520)	−0.007 to 0.013	0.054, −0.014	−0.204 (0.054)	−0.005, −0.300 (0.006) *	−0.009 to −0.001	0.133, 0.070	−0.127 (0.232)	−0.019, −0.035 (0.755)	−0.142 to 0.104	0.051, −0.018
	E velocity	0.209 (0.048) *	0.000, 0.115 (0.297)	−0.000199 to 0.000199	0.137, 0.075	0.259 (0.014) *	0.000, 0.241 (0.022) *	−0.002 to 0.006	0.179, 0.120	0.040 (0.711)	0.002, 0.113 (0.289)	−0.000199 to 0.000199	0.137, 0.075
	E/A ratio	−0.011 (0.915)	0.000, −0.049 (0.645)	−0.000199 to 0.000199	0.211, 0.154	−0.136 (0.202)	3.639 × 10^−5^, 0.038 (0.708)	−0.000163 to 0.000235	0.211, 0.154	−0.193 (0.068)	−0.003, −0.089 (0.379)	−0.009 to 0.003	0.217, 0.160
	Av E/e′	0.247 (0.019) *	0.002, 0.150 (0.177)	−0.002 to 0.006	0.127, 0.064	0.468 (<0.001) *	0.002, 0.274 (0.010) *	0.000 to 0.004	0.178, 0.118	0.261 (0.013) *	0.026, 0.152 (0.155)	−0.010 to 0.062	0.130, 0.067
	EDT	−0.008 (0.939)	0.027, 0.094 (0.352)	−0.031 to 0.085	0.287, 0.235	−0.056 (0.599)	−0.025, −0.215 (0.025) *	−0.047 to −0.003	0.322, 0.273	0.074 (0.486)	0.147, 0.041 (0.673)	−0.541 to 0.835	0.281, 0.229

* *p* < 0.05, significant. *r*, Spearman correlation coefficient; B, unstandardized coefficient of multiple linear regression; β, standardized coefficient of multiple linear regression.

## Data Availability

The study’s original contributions are documented within the article. For further inquiries, please contact the corresponding author directly.

## Abbreviations

The following abbreviations are used in this manuscript:

MMPs	Matrix metalloproteinases	LV	Left ventricular
MMP-2	Matrix metalloproteinase-2	LA	Left atrial
MMP-9	Matrix metalloproteinase-9	E/A	Ratio of E velocity to atrial velocity
TIMPs	Tissue inhibitors of metalloproteinases	E velocity	Early mitral inflow velocity
TIMP-3	Tissue inhibitor of metalloproteinase-3	ANOVA	One-way analysis of variance
CAD	Coronary artery disease	ANCOVA	Analysis of covariance
ACS	Acute coronary syndrome	SEM	Standard error of the mean
CCS	Chronic coronary syndrome	CHD	Coronary heart disease
LVEF	Left ventricular ejection fraction	CVD	Cardiovascular disease
EDT	E-wave deceleration time	BP	Blood pressure
ECM	Extracellular matrix	TNF-α	Tumour necrosis factor-alpha
CT	Computed tomography	IL-1β	Interleukin-1β
